# Perforated Viscus as a Rare Complication of Ovarian Hyperstimulation Syndrome: A Case Report

**DOI:** 10.7759/cureus.117851

**Published:** 2026-10-13

**Authors:** Hanan Alajrad, Doaa M Sabir, Duha Mohmmad, Rania El Hag, Zeinab Abd Elmohsin Mostafa

**Affiliations:** 1 Emergency Medicine, Hamad Medical Corporation, Doha, QAT; 2 Emergency Medicine, Sudan International University, Khartoum, SDN

**Keywords:** bariatric surgery complications, ivf therapy complications, marginal ulcer perforation, ovarian hyperstimulation syndrome (ohss), perforated hollow viscus

## Abstract

Ovarian hyperstimulation syndrome (OHSS) is a potentially serious complication of in vitro fertilization (IVF). While abdominal pain and intra-abdominal free fluid are common manifestations of OHSS, intestinal perforation occurring in patients with OHSS is extremely uncommon. We report a case of small bowel perforation following IVF in a patient with features of OHSS, presenting a diagnostic challenge due to overlapping clinical features.

A 39-year-old female patient presented with severe lower abdominal pain 5 days after undergoing ovum retrieval for IVF. The patient’s past surgical history included sleeve gastrectomy nine years prior and gastric bypass surgery two years earlier, without postoperative complications during the preceding two years. On presentation, she was tachycardic with lower abdominal tenderness and guarding. Laboratory investigations showed leukocytosis and a rising CRP. Bedside ultrasound showed intra-abdominal free fluid. IV contrast-enhanced CT raised concerns for gastrointestinal perforation and demonstrated features suggestive of OHSS. The patient rapidly deteriorated. Given the concern for gastrointestinal perforation and evolving septic shock, the patient underwent diagnostic laparoscopy, which was converted to a midline laparotomy. A 1.5-cm perforation at the jejunal omega loop near the gastrojejunal anastomosis was identified and primarily repaired with abdominal washout. During the laparotomy, a bulky uterus and enlarged ovaries with large ovarian cysts were also noted. The postoperative course was complicated by pulmonary embolism and septic shock; however, she improved with appropriate management and was discharged home in good condition.

This case emphasizes that although OHSS commonly presents with abdominal pain and ascites following IVF, physicians should consider bowel perforation as a differential diagnosis in patients presenting with severe abdominal pain, particularly in patients with previous bariatric surgery.

## Introduction

Ovarian hyperstimulation syndrome (OHSS) is a rare but potentially serious adverse outcome associated with controlled ovarian stimulation during assisted reproductive technology (ART) [1]. In the past, moderate-to-severe OHSS has been reported to occur in about 1%-5% of in vitro fertilization (IVF) cycles [1]. This condition mainly develops in patients who are highly responsive to exogenous gonadotropins after receiving human chorionic gonadotropin (hCG). The clinical presentation of OHSS ranges from mild symptoms to severe complications. Patients mainly present with ovarian enlargement, abdominal distention, and pain. Fluid shift from the intravascular compartment to the third space may result in ascites, pleural and pericardial effusions, and generalized edema. Life-threatening complications, such as acute respiratory distress syndrome, thromboembolism, and acute renal failure, can develop in severe OHSS [2]. 

Gastrointestinal perforation is an exceedingly rare complication reported in association with severe OHSS. To date, the available literature is limited to isolated case reports describing this complication in patients with severe or critical OHSS. Due to the scarcity of published evidence, a direct causal relationship between OHSS and gastrointestinal perforation remains unestablished [3-5].

We report a rare case of perforated viscus complicating severe OHSS, highlighting the importance of considering additional intra-abdominal surgical pathology in patients who develop worsening abdominal symptoms or unexpected clinical deterioration despite appropriate management of OHSS.

## Case presentation

A 39-year-old woman presented to the Emergency Department with a 1-day history of severe lower abdominal pain. The pain was sharp, radiated to the back and epigastric region, and was associated with nausea and vomiting. She denied fever, diarrhea, constipation, urinary symptoms, or any other associated complaints.

Five days prior to presentation, she had undergone IVF therapy (ovum pick-up without embryo transfer). Her past surgical history was significant for sleeve gastrectomy performed nine years earlier, gastric bypass surgery two years earlier, and two previous cesarean sections. Her medical history was notable for depressive disorder diagnosed seven years ago, for which she was maintained on amitriptyline, escitalopram, and flupenthixol. She had no other significant comorbidities.

On arrival, the patient appeared to be in severe distress and was screaming in pain. She was tachycardic with a heart rate of 140 beats/min, while her initial blood pressure, temperature, respiratory rate, and oxygen saturation were within normal limits. Abdominal examination revealed mild lower abdominal tenderness with guarding and mild rigidity, without rebound tenderness or palpable organomegaly. The remainder of the physical examination was unremarkable.

Initial management included intravenous analgesia with 15 mg of morphine administered in three divided doses, paracetamol, esomeprazole, and empirical ceftriaxone. Laboratory investigations were obtained. Bedside ultrasound demonstrated moderate free fluid within the abdomen and pelvis. The Gynecology team was consulted; however, they recommended excluding a surgical cause before assuming care. Consequently, a contrast-enhanced computed tomography (CT) scan of the abdomen and pelvis was requested. However, the patient could not tolerate the oral contrast; therefore, only IV contrast was given. The Bariatric Surgery team was also consulted but considered the presentation unlikely to be related to her previous bariatric procedures, given the absence of postoperative complications over the preceding two years following gastric bypass and nine years following sleeve gastrectomy.

Laboratory investigations demonstrated leukocytosis with a white blood cell count of 17 × 10⁹/L and a hematocrit of 47% (high). Her C-reactive protein (CRP) was initially 1.8 mg/L, but increased rapidly to 77 mg/L within a few hours and continued to rise to the highest value of 257 mg/L; subsequently, it started trending down to 85.3 mg/L upon discharge (Table 1).

**Table 1:** Laboratory investigations Results of the laboratory investigations preoperatively, two hours postoperatively, and before discharge, along with the normal range values.

Laboratory test	On arrival	2 hours postoperative	Upon discharge	Normal value
White blood cell count (WBCs)	17.4 x10^3/uL	5.1 x10^3/uL	16 x10^3/uL	4 - 10 x10^3/uL
Hemoglobin (Hb)	11.1 gm/dl	13 gm/dl	10.4 gm/dl	13 - 17 gm/dl
Platelets	485 x10^3/uL	357 x10^3/uL	411 x10^3/uL	150 – 410 x10^3/uL
Absolute neutrophil count (ANC)	11.9 x10^3/uL	3.1 4 x10^3/uL	10.8 x10^3/uL	2 - 7 x10^3/uL
C- reactive protein (CRP)	1.8 mg/L	77.2 mg/L	85.3 mg/L	0.0 - 5 mg/L
Beta HCG	30 mIU/ml	-------	3 mIU/ml	0 - 7 mIU/ml
Estradiol	6,100 pmol/L	-------	1790 pmol/L	45.4 - 854 pmol/L
Creatinine	46 umol/L	56 umol/L	33 umol/L	62 - 106 umol/L
Blood urea nitrogen (BUN)	3.5 mmol/L	2.9 mmol/L	13 mmol/L	2.5 - 7.8 mmol/L
Lipase	166 U/L	146 U/L	-------	13 - 60 U/L
Lactic acid	4.4 mmol/L	4.20 mmol/L	0.60 mmol/L	0.5 - 2.2 mmol/L
Procalcitonin	0.62 ng/ml	4.77 ng/ml	29.7 ng/ml	>2 indicates high risk for sepsis.
Aspartate aminotransferase (AST)	Hemolyzed	49 U/L	14 U/L	0 - 40 U/L
Alanine aminotransferase (ALT)	13 U/L	30 U/L	8 U/L	0 - 41 U/L
Alkaline phosphatase (ALP)	89 U/L	59 U/L	84 U/l	40 - 129 U/L
Total bilirubin	5 umol/L	22 umol/L	11 umol/L	0 - 21 umol/L
Potassium	3.5 mmol/L	5 mmol/L	4.2 mmol/L	3.5 - 5.3 mmol/L

Following that, preoperative contrast-enhanced CT of the abdomen and pelvis demonstrated extensive pneumoperitoneum, moderate free intraperitoneal fluid, and an apparent discontinuity of the posterior wall of the gastric remnant near the fundus, raising suspicion of gastric perforation. However, intraoperatively, the site of perforation was found to be at the jejunal omega loop near the gastrojejunal anastomosis. Hence, a discrepancy between the preoperative CT interpretation and the intraoperative findings was identified. In addition, bilateral enlarged ovaries with multiple follicles, moderate ascites, and pleural effusion were noted on CT, findings suggestive of OHSS (Figures 1, 2).

**Figure 1:**
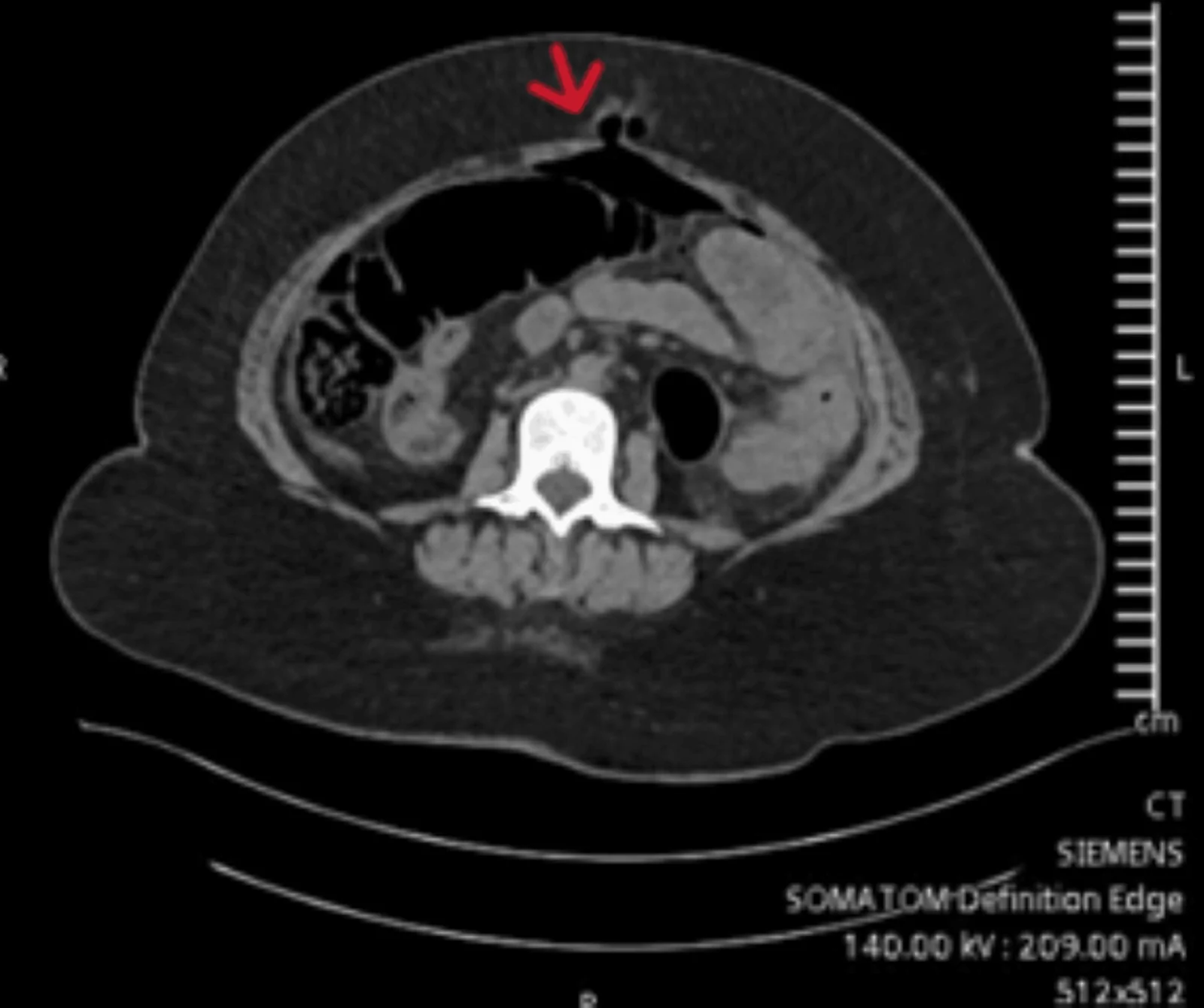
CT abdomen with IV contrast showing perforation of the stomach CT abdomen with IV contrast demonstrating perforation at the jejunal omega loop near the gastrojejunal anastomosis along with stranding and free fluids in the abdomen.

**Figure 2:**
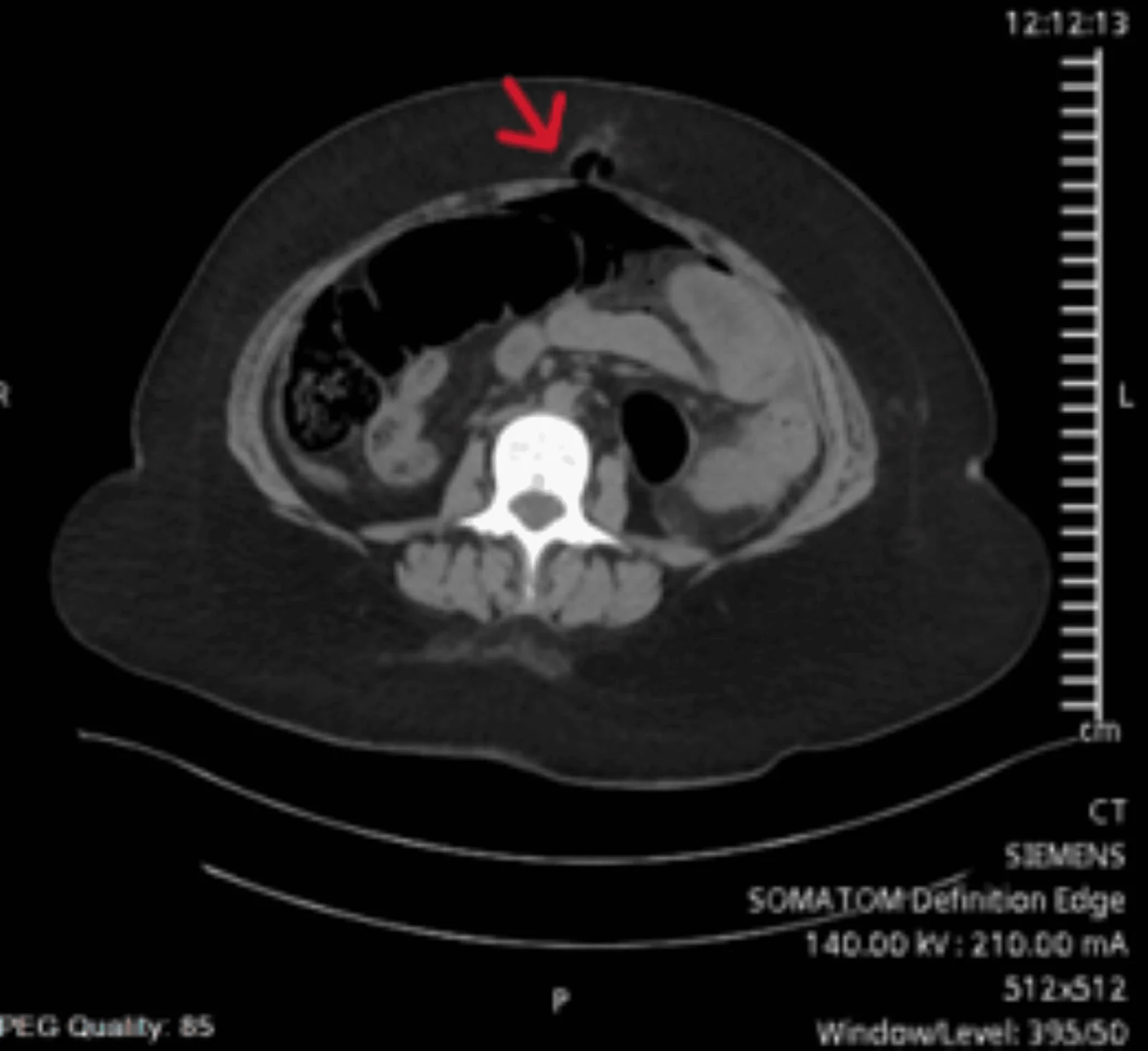
CT abdomen with IV contrast showing perforated viscus. Red arrow indicating perforation at the jejunal omega loop near the gastrojejunal anastomosis along with stranding and free fluids in the abdomen.

Despite ongoing management, the patient's condition deteriorated clinically. She developed worsening tachycardia (heart rate 154 beats/min), tachypnea (respiratory rate 41 breaths/min), and profound hypotension (59/49 mmHg), which responded to aggressive intravenous fluid resuscitation. She remained afebrile throughout. Her electrocardiogram (ECG) showed sinus tachycardia (Figure 3), and her chest radiograph showed bilateral consolidation (Figure 4).

**Figure 3:**
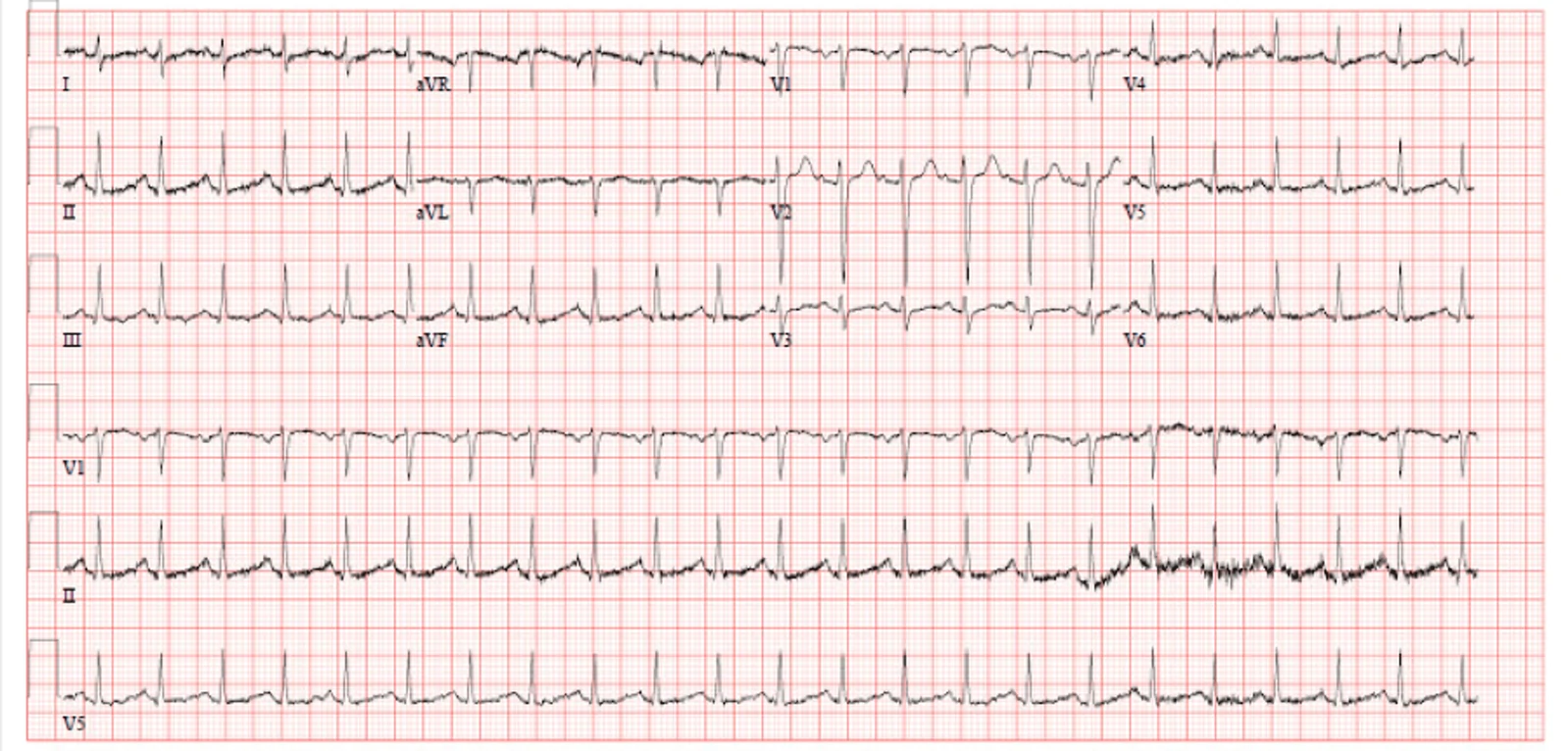
ECG Sinus tachycardia noted on the ECG

**Figure 4:**
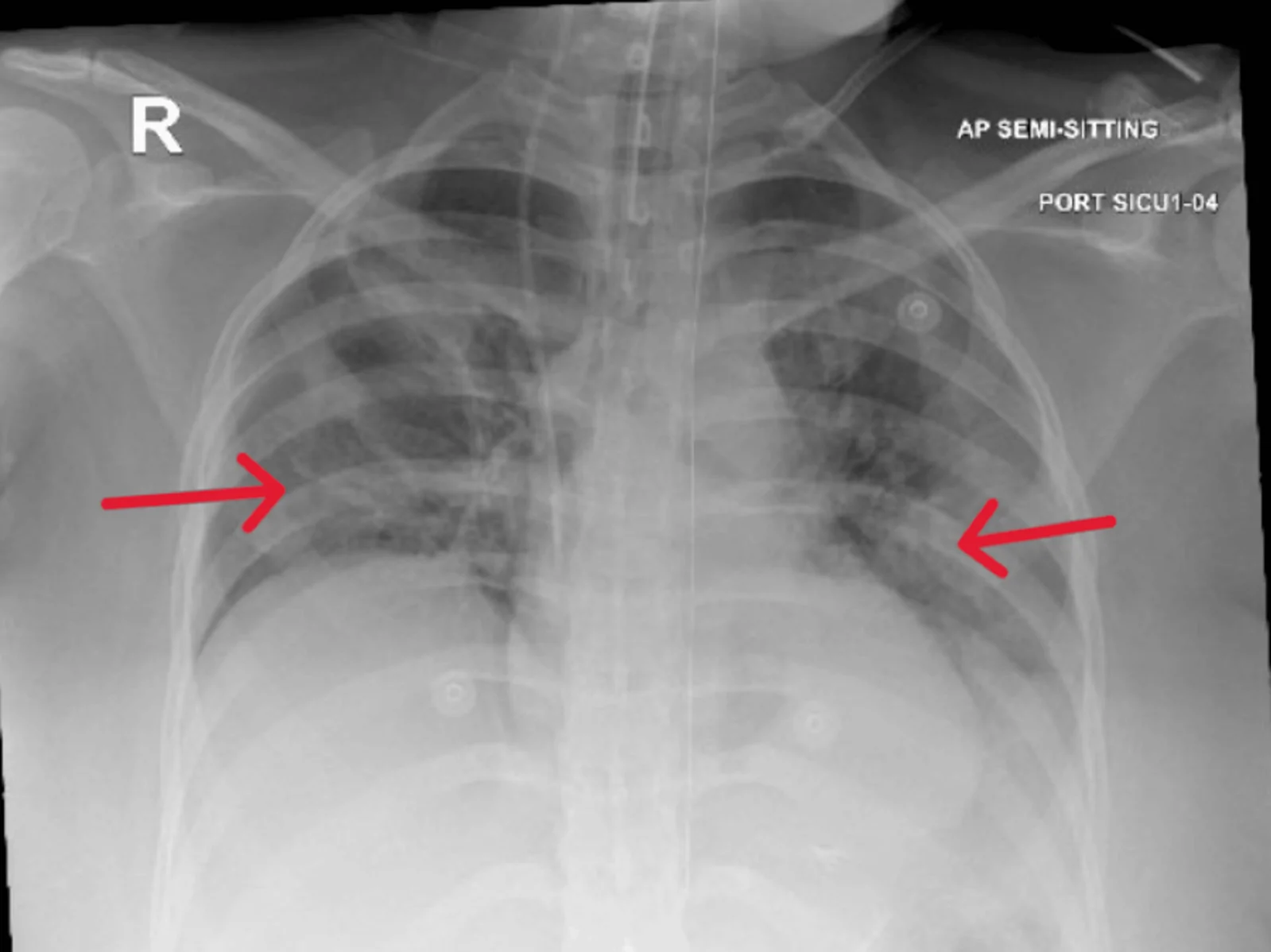
CXR Chest X-ray showing bilateral lung infiltrates with right-sided volume loss

Given the concern for gastrointestinal perforation and evolving septic shock, the patient was taken emergently to the operating theatre by the Acute Care Surgery (ACS) team. She underwent diagnostic laparoscopy, which was converted to a midline laparotomy, followed by primary repair of a small bowel perforation, abdominal washout, and abdominal closure. During the procedure, both the Bariatric Surgery and Gynecology teams were consulted intraoperatively. Operative findings included a gush of air upon entry into the abdomen, large amounts of bilious ascitic fluid predominantly within the upper quadrants, pyogenic membranes over the left upper quadrant, and a 1.5-cm perforation at the jejunal omega loop near the gastrojejunal anastomosis. The liver and appendix appeared normal. A bulky uterus and enlarged ovaries with large ovarian cysts were also noted.

Postoperatively, the patient was admitted to the Surgical Intensive Care Unit (SICU), where she remained intubated and mechanically ventilated for seven days. She was subsequently transferred to the bariatric surgery ward and was discharged home after an 11-day hospital stay. Her hospital course was complicated by the development of a right upper lobe pulmonary embolism (PE). On postoperative day 3, the patient demonstrated clinical deterioration, characterized by elevated inflammatory markers, tachycardia, a right lower respiratory tract infection, and a surgical site infection with wound discharge. Consequently, a repeat contrast-enhanced CT of the abdomen and pelvis, along with a CT of the thorax, was performed. Imaging confirmed the diagnosis of pulmonary embolism and excluded any evidence of a postoperative intra-abdominal collection (Figure 5). Therefore, PE was successfully managed with therapeutic anticoagulation using dalteparin. She also developed septic shock, requiring broad-spectrum antimicrobial therapy with piperacillin-tazobactam, later escalated to meropenem, along with vasopressor support, ventilatory support, and comprehensive intensive care management. Her blood and urine cultures did not grow any organism. As a result, the patient gradually improved clinically and was discharged home in good condition.

**Figure 5:**
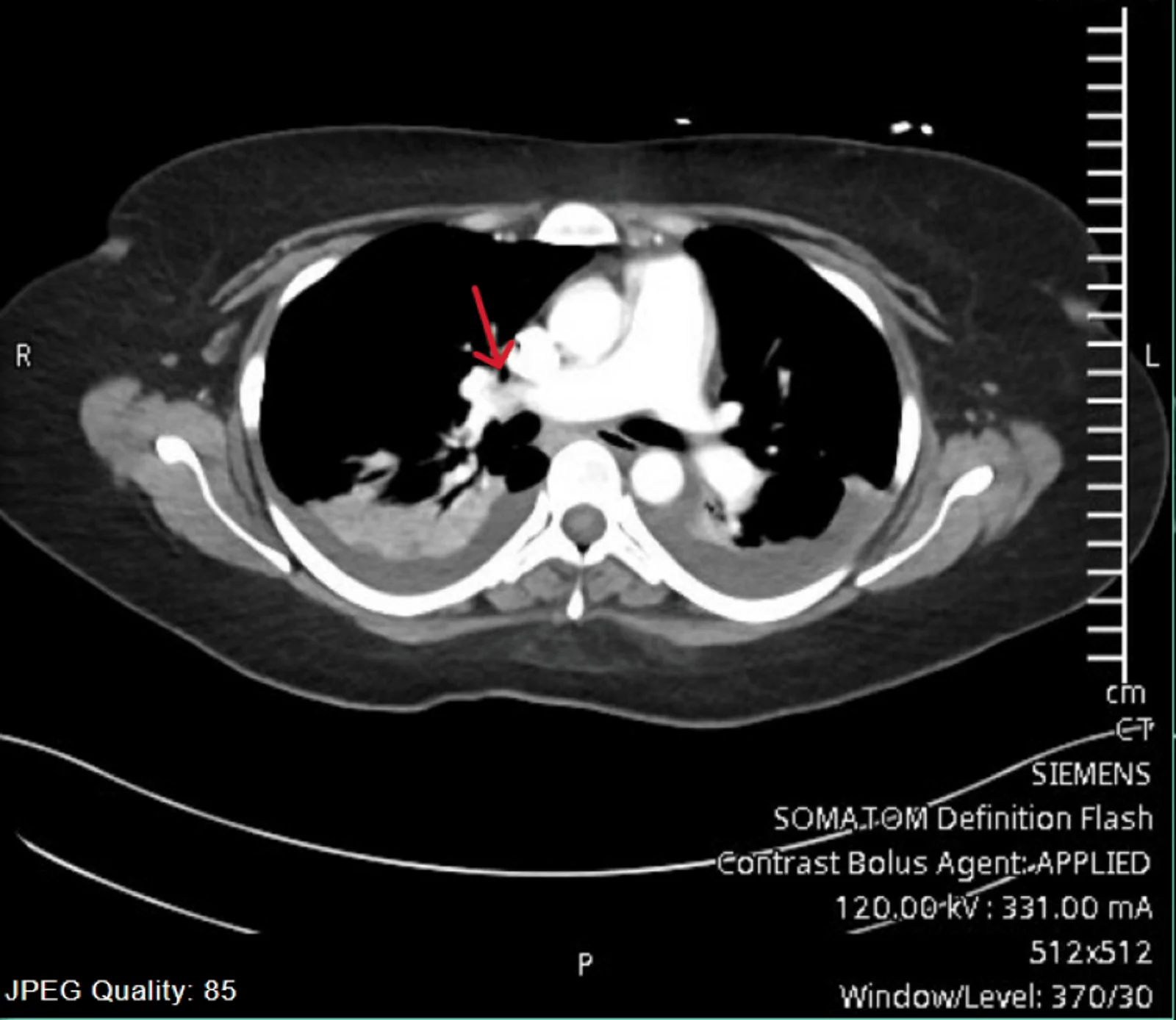
CT thorax showing PE Filling defect seen within the distal right upper lobe pulmonary artery, in keeping with pulmonary embolism (PE)

## Discussion

We presented a rare case of a giant perforated gastrojejunostomy ulcer occurring in a patient with severe OHSS. OHSS is a serious complication of controlled ovarian hyperstimulation during assisted reproductive technology that can potentially be life-threatening. It manifests as ovarian enlargement and increased vascular permeability, leading to the transfer of fluid from the intravascular compartment to the third space, particularly the abdominal cavity. Patients with severe OHSS may develop abdominal distension, ascites, vomiting, intravascular volume depletion, renal dysfunction, thromboembolic complications, and respiratory compromise [6,7].

In this case, the patient showed clinical features consistent with severe OHSS, including ascites, severe abdominal pain, abdominal distension, vomiting, and hemodynamic instability following an attempted in vitro fertilization procedure. Nevertheless, further evaluation revealed a perforated gastrojejunostomy anastomotic ulcer. Although abdominal pain, vomiting, ascites, and tachycardia may occur in severe OHSS, severe abdominal pain or clinical deterioration should prompt careful consideration of alternative or concurrent surgical pathology. The Royal College of Obstetricians and Gynecologists specifically recommends that other causes of symptoms be excluded in women with OHSS who present with severe abdominal pain [7].

The patient underwent laparoscopic sleeve gastrectomy in 2014, followed by a secondary omega-loop gastric bypass in 2019. Gastrojejunal ulcers, also known as marginal or anastomotic ulcers, are complications of gastric bypass procedures. They can occur months or years after surgery and often present with abdominal pain, vomiting, gastrointestinal bleeding, perforation, or fistula formation [8,9]. Although most marginal ulcers are managed medically, perforation is a rare but potentially life-threatening complication that generally requires urgent surgical management [8,9].

In this case, the diagnosis of a perforated anastomotic ulcer was initially challenging because the patient’s symptoms and bedside ultrasonography findings were compatible with severe OHSS. The presence of a large volume of abdominal and pelvic free fluid, together with bilaterally enlarged ovaries containing multiple cysts, supported the initial diagnosis of OHSS. However, these findings did not exclude a concurrent intra-abdominal surgical emergency. In patients with a history of bariatric surgery, persistent abdominal pain, vomiting, tachycardia, or tachypnea should not be attributed automatically to the previous surgical procedure or to another apparent diagnosis. Delayed recognition of acute abdominal complications after bariatric surgery may have serious or fatal consequences [10].

The patient’s previous bariatric procedures were therefore an important diagnostic consideration. Clinicians should remain aware that late complications involving the gastrojejunostomy may occur long after the original operation. The literature describes marginal ulceration as a multifactorial complication associated with factors such as smoking, nonsteroidal anti-inflammatory drug use, Helicobacter pylori infection, diabetes, ischemia, and technical features of the anastomosis [8,9]. The specific cause of ulceration in this patient could not be established from the available clinical information.

Contrast-enhanced CT, preferably with oral contrast when suitable, is the recommended diagnostic modality for acute abdominal pain in stable patients with previous bariatric surgery [10]. CT may demonstrate free intraperitoneal air, altered gastrointestinal anatomy, inflammatory changes around the gastrojejunostomy, extraluminal contrast, and other signs of perforation. In addition, plain abdominal radiography is an alternative rapid modality that can illustrate subdiaphragmatic free air; however, its diagnostic role is limited compared with CT [10,11].

Early involvement of both the Gynecology and Bariatric Surgery teams was essential in this case. The patient underwent laparoscopy, which was converted to a midline laparotomy, followed by repair of the perforated ulcer. Current guidance emphasizes prompt surgical exploration in unstable patients with peritonitis after bariatric surgery. Primary repair, with reinforcement using an omental patch when technically feasible, is an accepted approach for selected perforated marginal ulcers [10].

Although a direct causal relationship between OHSS and marginal ulcer perforation has not been established, several hypothetical mechanisms may explain their coexistence in this patient. OHSS-associated capillary leak, ascites, intravascular volume depletion, and hemoconcentration could potentially affect tissue perfusion and ulcer healing; however, these effects on marginal ulcers remain unproven. Pregnancy-related hormonal changes, including progesterone-mediated reductions in gastrointestinal motility, may also contribute to gastrointestinal symptoms but are not established causes of marginal ulcer formation, and our patient was not pregnant. Furthermore, OHSS-related abdominal pain, vomiting, distension, and leukocytosis may overlap with the presentation of ulcer perforation, potentially complicating recognition and delaying diagnosis. These proposed mechanisms should be interpreted cautiously, as the relationship between OHSS and perforated marginal ulcers remains uncertain [1,8,12].

To our knowledge, the coexistence of suspected severe OHSS and a perforated gastrojejunostomy ulcer following previous bariatric surgery is exceedingly rare. Marginal ulceration is a recognized complication of one-anastomosis gastric bypass (OAGB). Unlike Roux-en-Y gastric bypass, OAGB involves a single gastrojejunal anastomosis with potential exposure to biliary reflux. An OAGB-specific systematic review reported a pooled marginal ulcer incidence of approximately 2.6%, while comparative evidence suggests that symptomatic marginal ulcer rates may be similar following OAGB and RYGB. These findings support the relevance of marginal ulceration after OAGB, although evidence regarding its incidence and risk factors remains heterogeneous [13-15].

OHSS is associated with an increased risk of venous thromboembolism (VTE) due to hemoconcentration and prothrombotic changes. In this patient, suspected OHSS, emergency abdominal surgery, and postoperative inflammation may have contributed to the development of pulmonary embolism. Mechanical VTE prophylaxis was initiated on postoperative day 1, followed by dalteparin 5,000 units subcutaneously once daily on postoperative day 2. Despite prophylaxis, pulmonary embolism was confirmed on postoperative day 3, highlighting the importance of maintaining clinical vigilance in patients with multiple thromboembolic risk factors [7].

This case emphasizes considering a perforated anastomotic ulcer as a possible late complication of bariatric surgery in patients presenting with severe OHSS, especially when abdominal pain is disproportionate, progressive, or associated with hemodynamic deterioration. The case also stresses the importance of early multidisciplinary assessment and timely imaging or surgical exploration, particularly when the clinical course is not fully explained by OHSS alone.

## Conclusions

This case highlights a rare occurrence of gastrointestinal perforation in a patient with OHSS following IVF and a history of gastrointestinal surgery. It emphasizes the importance of considering gastrointestinal perforation in patients presenting with OHSS-like features and acute abdominal deterioration. Early multidisciplinary assessment and timely surgical evaluation should be considered in such cases to facilitate appropriate diagnosis and management.

## References

[1] (2026). Corrigendum to 'Prevention of moderate and severe ovarian hyperstimulation syndrome: a guideline' [Fertil Steril 2024;121:230-45]. Fertil Steril.

[2] Palomba S, Costanzi F, Nelson SM, Caserta D, Humaidan P (2023). Interventions to prevent or reduce the incidence and severity of ovarian hyperstimulation syndrome: a systematic umbrella review of the best clinical evidence. Reprod Biol Endocrinol.

[3] Uhler ML, Budinger GR, Gabram SG, Zinaman MJ (2001). Perforated duodenal ulcer associated with ovarian hyperstimulation syndrome: case report. Hum Reprod.

[4] Memarzadeh MT (2010). A fatal case of ovarian hyperstimulation syndrome with perforated duodenal ulcer. Hum Reprod.

[5] Fujimoto A, Osuga Y, Yano T, Kusumi M, Kurosawa T, Fujii T, Taketani Y (2002). Ovarian hyperstimulation syndrome complicated by peritonitis due to perforated appendicitis. Hum Reprod.

[6] Kumar P, Sait SF, Sharma A, Kumar M (2011). Ovarian hyperstimulation syndrome. J Hum Reprod Sci.

[7] Hamoda H, Drakeley AJ, Brian K, Evbuomwan IO, Mathur R (2026). The management of ovarian hyperstimulation syndrome green-Top Guideline No. 5. BJOG.

[8] Salame M, Jawhar N, Belluzzi A, Al-Kordi M, Storm AC, Abu Dayyeh BK, Ghanem OM (2023). Marginal ulcers after Roux-en-Y gastric bypass: etiology, diagnosis, and management. J Clin Med.

[9] Adduci AJ, Phillips CH, Harvin H (2015). Prospective diagnosis of marginal ulceration following Roux-en-Y gastric bypass with computed tomography. Radiol Case Rep.

[10] De Simone B, Chouillard E, Ramos AC (2022). Operative management of acute abdomen after bariatric surgery in the emergency setting: the OBA guidelines. World J Emerg Surg.

[11] De Simone B, Ansaloni L, Sartelli M (2020). The operative management in bariatric acute abdomen (OBA) survey: long-term complications of bariatric surgery and the emergency surgeon's point of view. World J Emerg Surg.

[12] Rosenberg H, Rangwala S, Magee B (2024). Ovarian hyperstimulation syndrome. CMAJ.

[13] Lee S, Supparamaniam S, Varghese C, Mahawar K (2023). Marginal ulcers following one-anastomosis gastric bypass: a systematic review and meta-analysis. Obes Surg.

[14] Kupietzky A, Dodi O, Cohen N (2024). Similar rates of symptomatic marginal ulcers after one-anastomosis-gastric bypass compared to Roux-en-Y gastric bypass. Obes Surg.

[15] Shahabi Shahmiri S, Mehraz A, Jazi K, Vakili K, Esparham A, Kermansaravi M (2025). Mapping the landscape of marginal ulcers after one anastomosis gastric bypass: incidence, regional trends, and treatment insights from a systematic review and meta-analysis. Obes Surg.

